# Weathering the storm: diagnosis and treatment of a life-threatening disseminated *Nocardia otitidiscaviarum* infection

**DOI:** 10.3389/fcimb.2024.1397847

**Published:** 2024-05-31

**Authors:** Li-Yan Zhang, Liang Wang, Zeeshan Umar, Yuan-Hong Huang, Bing Gu

**Affiliations:** ^1^ Laboratory Medicine, Ganzhou Municipal Hospital, Guangdong Provincial People’s Hospital Ganzhou Hospital, Ganzhou, Guangdong, China; ^2^ Laboratory Medicine, Guangdong Provincial People’s Hospital (Guangdong Academy of Medical Sciences), Southern Medical University, Guangzhou, Guangdong, China; ^3^ Centre for Precision Health, School of Medical and Health Sciences, Edith Cowan University, Perth, WA, Australia

**Keywords:** *Nocardia otitidiscaviarum*, nocardiosis, minimal change disease, microscopic examination, mass spectrometry, metagenomic sequencing

## Abstract

Nocardiosis demonstrates a temporal categorization that includes acute, subacute, and chronic stages alongside distinct typical localizations such as pulmonary, cutaneous, and disseminated forms. Disseminated nocardiosis, commonly caused by *Nocardia asteroides*, *N. brasiliensis*, and *N. farcinica*, continues to result in substantial morbidity and mortality. Herein, we report a life-threatening disseminated nocardiosis caused by *Nocardia otitidiscaviarum* in a patient with minimal change disease. This study emphasizes the difficulty in the diagnosis and treatment of unknown infections in clinical settings and highlights the important role played by laboratories in solving infectious diseases caused by rare pathogens.

## Introduction

Nocardiosis in humans was first reported in Vienna, Austria, by Eppinger in 1890 in a man with pulmonary disease with “pseudotuberculosis” of lungs and pleura (Eppinger, 1890), as well as the presence of caseous peribronchial lymph nodes, meningitis, and multiple brain abscesses (Lee et al., 2021). The disease is caused by a group of opportunistic bacterial pathogens belonging to the genus *Nocardia* that are slow-growing, Gram-variable, partially acid-fast, with filamentous branching and environmental ubiquity (Sah et al., 2020). *Nocardia* enters the human body via the respiratory tract (pulmonary infection) or skin (superficial cutaneous and subcutaneous infection) and usually causes damage in immunocompromised hosts (Sah et al., 2020). Of all *Nocardia* species, *Nocardia otitidiscaviarum* (formerly *N. caviae*) is a rarely reported pathogen with less known pathogenicity and incidence than other *Nocardia* species such as *N. asteroides*, *N. brasiliensis*, and *N. farcinica* (Clark et al., 1995; Sah et al., 2020). In a recent epidemiological study, it was found that among the 441 non-repetitive *Nocardia* strains reported in China from 2009 to 2021, only 26 strains (5.9%) were identified as *N. otitidiscaviarum* (Wang et al., 2022). Similarly, in Australia, the isolation percentage of *N. otitidiscaviarum* was reported to be 4.9% (Georghiou and Blacklock, 1992). Additionally, Beaman et al. reported that 1 out of 347 *Nocardia* isolates (2.9%) was *N. otitidiscaviarum* in the United States from 1972 to 1974 (Beaman et al., 1976). In another study by Kageyama et al., 14 out of 303 pathogenic *Nocardia* strains (4.62%) were identified as *N. otitidiscaviarum* from 1992 to 2001 in Japan (Kageyama et al., 2003).

*N. otitidiscaviarum* was initially identified from the middle ear infection of a guinea pig and reported by Snijders as a novel species in 1924 (Snijders, 1924; Sah et al., 2020). However, the first human infection was not reported until 1974, when two fatal systematic nocardiosis infections were recorded (Causey et al., 1974). Currently, it is still very challenging to make an early diagnosis of *Nocardia* infections in clinical settings. Because of the insufficient specificity of clinical features and strict requirement of laboratory detection, a 2- to 3-week duration could be required from specimen collection to *Nocardia* identification (Li et al., 2021).

To get a better understanding of nocardiosis infection caused by *N. otitidiscaviarum*, we reviewed several representative studies published recently, which investigated the infection by the bacterial pathogen with a focus on epidemiology, drug resistance, and detection. In particular, Wang et al. report on the species distribution and antimicrobial susceptibility of 441 *Nocardia* strains collected from various regions in China over 13 years, among which *N. farcinica* was the most commonly isolated species, primarily from lower respiratory tract specimens while *N. otitidiscaviarum* represented 5.9% of isolates, with the majority obtained from the lower respiratory tract (Wang et al., 2022). Interestingly, all strains of *N. otitidiscaviarum* were susceptible to linezolid, amikacin, and trimethoprim-sulfamethoxazole (TMP-SMX), highlighting the importance of accurate species identification and antibiotic susceptibility testing for effective management of nocardiosis (Wang et al., 2022). Different from the epidemiological study, drug-resistant *N. otitidiscaviarum* is not uncommon and can often cause serious infections, which have been frequently reported in clinical cases. For example, Barry et al. underscore the significance of considering *N. otitidiscaviarum* in at-risk patients with relevant occupational exposure, while highlighting the TMP-SMX resistance and the importance of suspecting it when clinical response is lacking, which may have significant implications for clinical management of similar infections (Barry et al., 2022). In addition, Saksena et al. present two cases of fatal pulmonary infection caused by the rare *N. otitidiscaviarum* in elderly patients (Saksena et al., 2020). Both cases exhibited drug resistance, particularly to TMP-SMX, despite empirical treatment with it, while the isolates were susceptible to amikacin, linezolid, ciprofloxacin, and gentamicin (Saksena et al., 2020). In addition, Ranjan reported that, in a case of pleural nocardiosis in a 38-year-old man with immune thrombocytopenia (ITP) and HIV, pleural fluid analysis revealed *N. otitidiscaviarum* growth resistant to multiple antibiotics but susceptible to amikacin, linezolid, and levofloxacin; despite aggressive treatment, including steroid therapy for ITP, the patient succumbed to sepsis and concurrent infections with *Candida guilliermondii*, *Escherichia coli*, and *Stenotrophomonas maltophilia*, underscoring the challenge of managing nocardiosis in immunocompromised individuals (Ranjan et al., 2024).

In early 2024, several case reports have described the rare infection by the bacterial pathogen *N. otitidiscaviarum*, indicating that more and more researchers and clinical doctors pay attention to this bacterial infection. For example, Srivastava and colleagues reported *N. otitidiscaviarum* causing pulmonary nocardiosis in India (Srivastava et al., 2024), while Lin et al. reported a pulmonary co-infection with *N. otitidiscaviarum* and *Aspergillus* (Lin et al., 2024). In another study, Erbaş et al. reported a rare case of a newborn with branchial cleft cyst infection due to *N. otitidiscaviarum* (Erbaş et al., 2024). In addition, two studies reported multidrug-resistant *N. otitidiscaviarum* causing fatal pleural nocardiosis in an immunosuppressed patient (Ranjan et al., 2024) and empyema thoracis in an elderly patient (Pérez Ramos et al., 2023), strengthening that special attention should be paid to both bacterial drug resistance and vulnerable populations (Zhang et al., 2023).

Taken together, early identification of *Nocardia* to species level, facilitated by mass spectrometry (MS), is crucial for improving treatment outcomes, particularly in critically ill patients, emphasizing the need for integrating MS into diagnostic algorithms for nocardiosis to guide appropriate therapy. In addition, the necessity of initiating combination therapy, including trimethoprim/sulfamethoxazole, with the assistance of tests of drug susceptibilities, is important, as resistance patterns differ among species, potentially leading to fatal outcomes without precision treatment.

In this brief research report, we present a rapid diagnosis and successful treatment of a life-threatening disseminated nocardiosis infection caused by *N. otitidiscaviarum* in a patient with minimal change disease (MCD) who underwent long-term hormone therapy. This case strengthens the important role laboratory medicine equipped with comprehensively analytical techniques plays in solving a clinical mystery caused by an unusual pathogen.

## Case presentation

A 66-year-old Chinese man was initially diagnosed with nephrotic syndrome due to proteinuria in the 1st Affiliated Hospital of Gan’nan Medical College in 2019. Because of the recurrent proteinuria symptom, the patient was admitted to Ganzhou Municipal Hospital in July 2020, where he was diagnosed with MCD via percutaneous biopsy. The patient received long-term, high-dose prednisone treatment to avoid the recurrence of proteinuria. On 1 December 2021, the patient presented with a worsening cough, thick yellow-green sputum, right scrotal swelling, and bilateral testicular redness without inducement. On 6 December, the patient was diagnosed with inflammatory changes in the right epididymis and right testicular hydrocele in Longnan County People’s Hospital and was given oral amoxicillin but failed to improve and started to show worsening symptoms.

The patient was then admitted to Ganzhou Municipal Hospital for hospitalization. On 8 December, the medical laboratory reported critical values of white blood cell count at 31.71 × 10^9^/L and a neutrophil count at 30.13 × 10^9^/L, indicating a serious infection despite unknown sites and reasons. Pain in the swollen epididymis rules out testicular torsion, abscess, scrotal hydrops, seminal cyst, hernia, trauma, and testicular cancer. Color ultrasound suggested that the inflammatory changes of the right epididymis and hydrocele of the right testis should be considered. Amoxicillin capsules were taken orally, but the testicular swelling and pain did not improve significantly, and the symptoms gradually worsened. Clinical symptoms of epididymitis include pain, swelling, and severe scrotal pain, which are often unilateral. Therefore, the patient was finally diagnosed with epididymitis, received ceftriaxone for anti-infective therapy, and was transferred to the urology department for further observation. On 9 December, the patient was given Sulperazon due to an unknown fever (38.7°C) and durative right scrotal pain. On physical examination, it was found that the patient had rales in the left lower lung, and weakened breath sounds in both lower lungs. Computed tomography (CT) showed infiltrative consolidation with cavitation, which suggested pneumonia. Finally, the multidisciplinary team (MDT) diagnosis recommended the transfer of the patient to the respiratory department for pulmonary infection treatment.

On 13 December, the patient developed a high fever and breathing difficulty with 90% blood oxygen saturation. Physical examination revealed that the skin on the pulp of the little finger was black and fluctuating, and multiple pus spots were scattered on the whole body with local skin redness. Chest CT showed an unknown infection in the upper lobes of both lungs and the lower lobe of the left lung. To identify the pathogenic bacteria, the MDT consisting of respiratory physicians, pharmacists, medical laboratory specialists, and medical imaging experts from Guangdong Provincial People’s Hospital was invited to investigate the case. Specimens of skin lesions were collected from the bedside for a 3-day culture inoculation on Columbia blood agar plates at 35 ± 2°C and smear microscopic examination. Combined with the patient’s medical history, laboratory tests, and chest CT, preliminary diagnosis was given as bacterial and fungal co-infection in bilateral lungs, which was accompanied by systemic spread. Further brain magnetic resonance imaging (MRI) was recommended, which confirmed intracranial infection. In particular, *Nocardia* culture in cerebrospinal fluid was positive. Head MRI showed space-occupying lesions. Clinical manifestations were worsening for consciousness, fever, and atypical clinical symptoms. No intracranial hypertension and no headache were reported. On 15 December, VITECK^®^ MS (bioMérieux, France) confirmed that the pathogenic bacterium was *N. otitidiscaviarum* with 99.9% confidence. Targeted next-generation sequencing (tNGS) was conducted on bronchoalveolar lavage fluid (BLF) on 16 December and the results came back on 17 December, in which two bacterial pathogens *N. otitidiscaviarum* (9×10^4^ copies/mL) and *Klebsiella aerogenes* (<100 copies/mL) were reported. For specific information and general procedures of the tNGS analysis, please refer to the **Supplementary Materials** . The tNGS result matched with clinical symptoms and the MS result. Taking all the evidence together, the final diagnosis of this intriguing case was disseminated nocardiosis.

**Figure 1:**
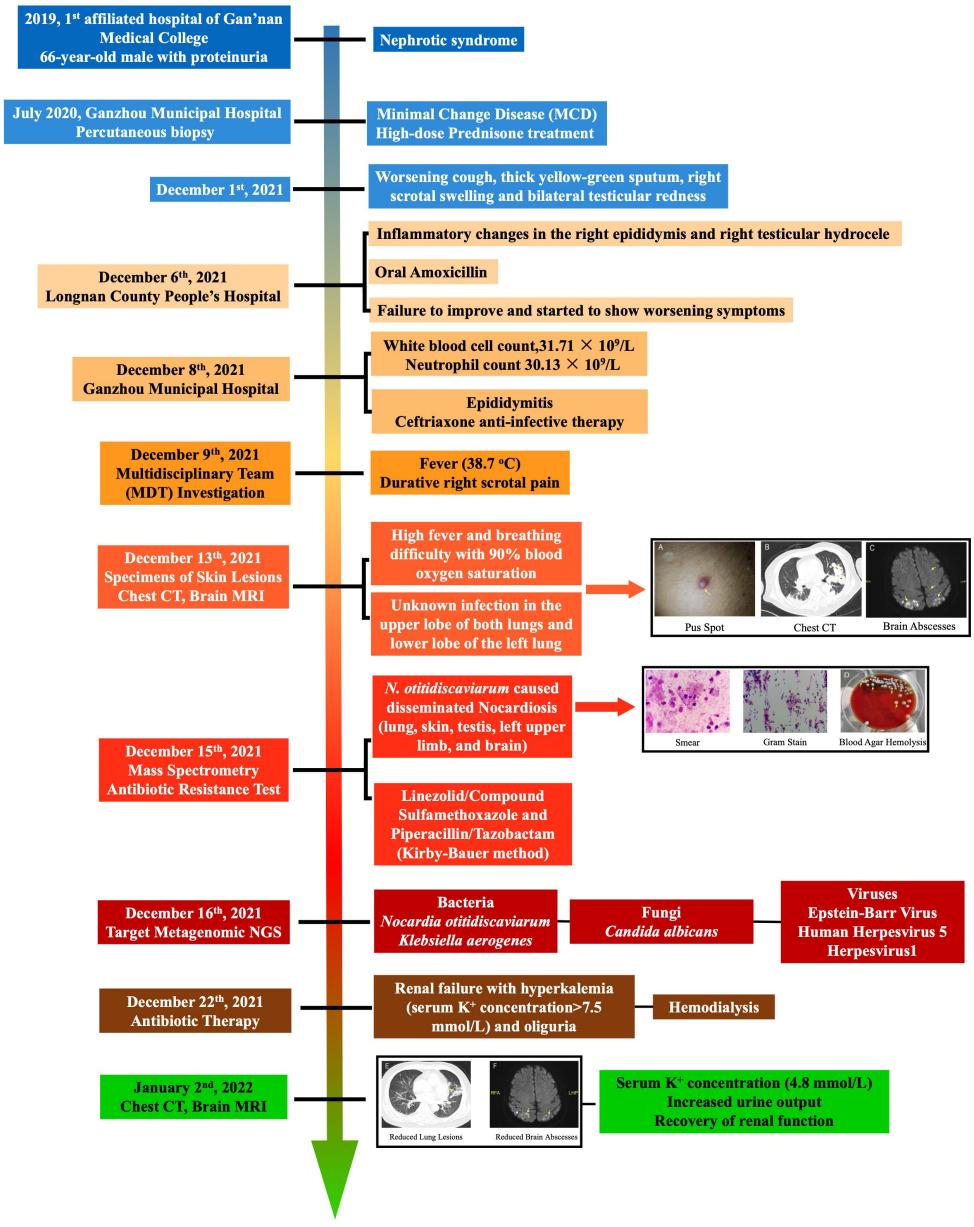
Timeline of clinical events summarizing the diagnosis and treatment of the patient from hormone therapy to infection presentation and eventually to treatment regimen. Enlarged images of pub spot, chest CT, smear and Gram stain slides, blood agar plates, and brain abscesses were available in the **Supplementary Materials**.

Susceptibility testing was conducted via the Kirby–Bauer method. Since the patient had underlying kidney disease, hence vancomycin intolerance, the combined therapy of linezolid/compound sulfamethoxazole for *N. otitidiscaviarum* and piperacillin/tazobactam for *K. aerogenes* was adopted for disseminated nocardiosis. It is noteworthy that on 22 December, during anti-infective therapy, the patient developed renal failure with hyperkalemia (serum potassium concentration >7.5 mmol/L) and oliguria, and also bore the risk of cardiac arrest at any time. Because the drug effect of potassium excretion was poor, the patient was treated with emergency hemodialysis. On 2 January 2022, the serum potassium concentration of the patient returned to 4.8 mmol/L with increased urine output and recovery of renal function. For a schematic illustration of the timeline of clinical events, please refer to **Figure 1**.

## Discussion

Infection with *N. otitidiscaviarum* is rarely reported in China and worldwide, making its diagnosis extremely difficult since it is less likely to be suspected during infection investigation (Liu et al., 2017; Zheng, 2019). Cutaneous infection by *N. otitidiscaviarum* is almost indistinguishable from skin diseases by common pyogenic organisms, which can seriously delay the diagnosis and treatment procedures (Clark et al., 1995). In this case, although we confirmed the infectious agent as *N. otitidiscaviarum* and successfully eradicated the bacterial pathogen via a timely antibiotic treatment, there were still a series of unanswered questions about when, where, and how the patient acquired the rare bacterium due to the very low prevalence of *N. otitidiscaviarum* in the environment when compared with other *Nocardia* species and other opportunistic pathogens (Liu et al., 2017). Since nocardiosis normally develops in immunocompetent and immunosuppressed patients and individuals receiving long-term or large-dose corticosteroid therapy (Zheng, 2019), it was well understandable that the patient with MCD in this study was susceptible to the infection of *N. otitidiscaviarum* due to long-term prednisone usage. During the diagnosis of the infection, non-typical clinical features also made it difficult to determine the infectious cause. A variety of traditional and advanced analytical techniques in the medical laboratory, such as culture, smear, MS, and metagenomics, were conducted to confirm the infectious agent rapidly and accurately, which led to the discovery of *N. otitidiscaviarum* infection in this case. Therefore, applying novel techniques and combining them with traditional methods for clinical diagnosis of infrequent infectious diseases is crucial. It is also worth emphasizing that, after confirming the causal pathogens, rapid determination of bacterial antibiotic resistance was also essential in the efficient treatment of the rare infection, especially when the patient had underlying diseases. In sum, we demonstrated a rare disseminated nocardiosis case caused by *N. otitidiscaviarum* in a patient with MCD with long-term hormone therapy, which showed that nocardiosis could present in various manners and involve multiple organs. According to the report, to achieve the early identification of the causative species and provide an appropriate treatment regimen for *Nocardia* infection, possible risk factors of the disease should be recognized, and the application of advanced analytical techniques is crucial.

## Data availability statement

The raw data supporting the conclusions of this article will be made available by the authors, without undue reservation.

## Ethics statement

The studies involving humans were approved by Ganzhou Municipal Hospital (Approval No. 2022041H). The studies were conducted in accordance with the local legislation and institutional requirements. The participants provided their written informed consent to participate in this study. Written informed consent was obtained from the individual(s) for the publication of any potentially identifiable images or data included in this article.

## Author contributions

LW: Investigation, Methodology, Validation, Visualization, Writing – original draft, Writing – review & editing. L-YZ: Data curation, Formal analysis, Investigation, Methodology, Validation, Visualization, Writing – original draft, Writing – review & editing. ZU: Formal analysis, Methodology, Validation, Visualization, Writing – original draft, Writing – review & editing. Y-HH: Formal analysis, Investigation, Methodology, Validation, Visualization, Writing – original draft, Writing – review & editing. BG: Conceptualization, Funding acquisition, Methodology, Project administration, Resources, Supervision, Validation, Writing – original draft, Writing – review & editing.
