# Supplementary material for: Weathering the storm: diagnosis and treatment of a life-threatening disseminated Nocardia otitidiscaviarum infection
Source: Front Cell Infect Microbiol. 2024 May 31;14:1397847. doi: 10.3389/fcimb.2024.1397847 (PMC11179430; doi:10.3389/fcimb.2024.1397847)
Supplement: Supplementary file 1 [file DataSheet_1.pdf]

**Title**

Weathering the storm: diagnosis and treatment of a life-threatening disseminated *Nocardia otitidiscaviarum* infection

**Running Title**

Nocardiosis in a Minimal Change Disease Patient

Li-Yan Zhang[1,2,†], Liang Wang[2,3,†,*], Zeeshan Umar[2], Yuan-Hong Huang[1], Bing Gu[2,*]

[1]Laboratory Medicine, Ganzhou Municipal Hospital, Guangdong Provincial People's Hospital Ganzhou Hospital, Ganzhou, Guangdong Province, China

[2]Laboratory Medicine, Guangdong Provincial People's Hospital (Guangdong Academy of Medical Sciences), Southern Medical University, Guangzhou, Guangdong Province, China

[3]Centre for Precision Health, School of Medical and Health Sciences, Edith Cowan University, Perth, Western Australia, Australia

[†]These authors contributed equally to this work.

[*]Correspondence:

Dr. Bing Gu, Laboratory Medicine, Guangdong Provincial People's Hospital (Guangdong Academy of Medical Sciences), Southern Medical University, Guangzhou, Guangdong Province, China (gubing@gdph.org.cn)

Dr. Liang Wang, Laboratory Medicine, Guangdong Provincial People's Hospital (Guangdong Academy of Medical Sciences), Southern Medical University, Guangzhou, Guangdong Province, China (wangliang@gdph.org.cn)

**Supplementary Material** Specific information and general procedures about the targeted NGS

Pathogeno One$^{TM}$ pathogen screening project was used in this study and conducted by Wuhan Kangsheng Genomic Medical Laboratory Co., Ltd. Utilizing Gensizer$^{TM}$ targeted sequencing technology, it identifies suspected pathogens in samples, covering hundreds of clinically relevant bacterial pathogenic microorganisms. The Gensizer$^{TM}$ technology has a minimum detection limit of 50 copies/mL for pathogenic microorganisms; however, microbial content below this limit may not be reliably detected. To enhance detection rates, it is recommended to collect samples from infection sites nearby, and if using blood samples, preferably before anti-infective treatment. Sampling should strictly adhere to standard procedures, with attention to cold storage. This testing is based on existing pathogen genomes or gene databases for probe design. It cannot exclude the possibility of missed detection due to complex mutations in pathogens carried by the individual, leading to inefficient or ineffective probe capture. If antibiotic-resistant genes are reported, it is uncertain whether they originate from a pathogenic organism. Furthermore, antibiotic resistance is related to gene expression and requires comprehensive consideration of clinical characteristics for final treatment decisions. The responsibility of the test is limited to the sample provided, and sampling from lesion areas is advised. Failure to include the pathogenic organism in the sample site or low pathogen concentration may result in missed detection. Additionally, due to the inability to guarantee a 100% sterile sampling environment, detection of environmental pathogens may occur, requiring comprehensive clinical judgment based on test results.
